# LONG-TERM EFFECTS OF EDUCATION ON LATE-LIFE DEPRESSION OF RURAL WOMEN IN CHINA

**DOI:** 10.1093/geroni/igad104.2876

**Published:** 2023-12-21

**Authors:** Lynn Hu

**Affiliations:** Pardee RAND Graduate School, Santa Monica, California, United States

## Abstract

Despite the noted association between educational attainment and mental health, there is considerable debate on whether this relationship is causal. Up to now, there are only a few studies using credible estimation strategies to examine the causal impact of education on mental health, and almost no evidence exists for low and middle-income developing countries from regions like Asia. This is concerning given that mental health problems and late-life depression are growing in the Asian population, especially for women and rural residents. In this paper, I aim to fill the gap by examining the long-term causal effect of additional educational attainment on the depressive symptoms of rural women in China. Using Harmonized CHARLS data, I leveraged the sudden end of the Chinese Cultural Revolution in 1976 as an exogenous shock to education, and employed both event study and regression discontinuity designs to estimate its causal effect on late-life depressive symptoms. Results show a protective effect of education for the rural women who stayed in rural areas, and a negative impact of education on the urbanized rural women. The impacts are likely to be mediated by childhood adversities (being poor and unhealthy), marriage status (being widowed), and disability status (difficulty in performing activities necessary for independent living). The proposed research will be the first study to examine the causal effect of education on the mental health of the Chinese population. It will add to the existing literature by providing evidence from underdeveloped regions in Asian countries and offering insights for future policymaking.

